# Scientific Items

**Published:** 1878-12-20

**Authors:** 


### *Scientific Items.*

### DRAPER'S SCIENTIFIC MEMOIRS.

The name of Dr. John Draper will hold a place in the history of American scientific investigation with those of Franklin, Rumford and Henry. For forty years past he has been a constant and devoted student of physical phenomena, and he has just gathered into a single volume (published by the Harpers) those of his scientific papers which deal with the subject of radiant energy. It is an octave of nearly 500 pages, and is entitled "Scientific Memoirs: Being Experimental Contributions to a Knowledge of Radiant Energy." It was for his work in this field that the American Academy of Science awarded him the Rumford Medal in 1875. Even to those not unfamiliar with Dr. Draper's position as an investigator, the book will give new ideas of the immense amount of original work he has done. It would take a long article merely to enumerate the instances in which he has been either the first or among the first in the great discoveries of a period remarkable for scientific progress, or in the application of these discoveries to practical purposes. He was the first on this side of the Atlantic to follow up Fraunhofer's study of the solar spectrum by finding new lines and showing that they could be photographed. In like manner he carried the work of Daguerre further than the Frenchman had dreamed of doing, by taking the first photographic portrait of a living subject. He was also the first to obtain a photograph of the moon, which Daguerre had attempted in vain. He showed that the decomposition of carbonic acid by plants was accomplished by the yellow rays of light, and not, as had been snpposed, by the violet. He also disproved the current notions as to the distribution of heat and chemical energy in the spectrum by showing that all the colored spaces were equally warm, and that every ray, and not the violet alone, can produce chemical effects. These are but a few out of the many important researches of Dr. Draper, hitherto accessible only in files of the higher scientific journals, or in the transactions of learned societies, but now collected in this interesting volume and brought within the reach of every student.—*Journal of Chemistry.*

### EDISON.

Edison, the great inventor, has a labortory in which he is constantly engaged in scientific experiments. He has many now in progress—"The action of chemicals upon various substances or upon each other, or the phenomena of substances subjected to the various forces at command. Strips of ivory, for instance, in a certain oil in six weeks become transparent. A globule of mercury in water, then with a little potassium added, takes various shapes for the opposite poles of the battery, retires coquettishly or is attracted, forms in whirlpools, changes color, or becomes immobile. There is no use at once for these results, but they are recorded in voluminous note-books. When the proper time comes they are borne in mind; some one of them may form the connecting link in the chain of an invaluable discovery. Then perhaps he tests for the thousandth time his carbon telephone for new perfections, and then goes on carrying forward a step each of the works in progress, or becomes wholly engrossed, according to his mood, in one."

Evolution.—Dr. Koegel assures the world of anthropologists that he has seen and examined men with tails in the Sunda Islands, specially among the Dajaks and in the Moluccas.

